# E3 ubiquitin ligase TRIM47 promotes intrahepatic cholangiocarcinoma progression by ubiquitinating fumarate hydratase and modulating macrophage polarization

**DOI:** 10.1016/j.jbc.2025.111035

**Published:** 2025-12-09

**Authors:** Yanyang Fu, Jixing Wu, Jian Shi, Changjun Jia

**Affiliations:** Department of General Surgery, Shengjing Hospital of China Medical University, Shenyang, People's Republic of China

**Keywords:** intrahepatic cholangiocarcinoma, tripartite motif containing 47, fumarate hydratase, fumarate, M2 macrophage polarization

## Abstract

To explore potential biomarkers in the progression of intrahepatic cholangiocarcinoma (ICC), the gene expression profiles in ICC and paired normal tissues were analyzed using mRNA sequencing. E3 ubiquitin ligase tripartite motif containing 47 (*TRIM47*) was found highly expressed in ICC tissues (log_2_ fold change = 2.473, *p* < 0.001), which was also observed in seven ICC samples compared with paired normal tissues. Patients with higher *TRIM47* expression exhibited shorter disease-free survival period. Functional studies indicated that TRIM47 knockdown in ICC cells suppressed cell growth in *vitro* and the tumorigenicity of tumor cells *in vivo*, whereas TRIM47 overexpression had the opposite effect. Furthermore, after coculture of TRIM47-silenced ICC cells and macrophages, the macrophages showed a reduction in M2 polarization bias. Cancer cells with TRIM47 downregulation were sensitive to macrophage cytotoxicity. Notably, the secretion of fumarate was reduced in TRIM47-silenced cancer cells, and the treatment of dimethyl fumarate, a derivative of fumarate, reversed the effect of TRIM47 downregulation on M2 polarization in macrophages. Through immunoprecipitation–LC/MS analysis, we obtained 1166 binding partners of TRIM47 in ICC cells. Among those partners, fumarate hydratase (FH), a catalytic enzyme for fumarate metabolism, was bound with TRIM47. Knockdown of TRIM47 increased the protein half-life of FH from 1.33 h to 2.25 h. Overexpression of TRIM47 reduced the protein expression of FH and increased its ubiquitination. FH overexpression restored the effects of TRIM47. Collectively, the observations demonstrate that TRIM47 accelerates the progression of ICC by interacting with FH and ubiquitinating FH, indicating that the TRIM47–FH axis may be a potential target for ICC treatment.

Intrahepatic cholangiocarcinoma (ICC) is the second most common primary liver cancer, following hepatocellular carcinoma (HCC), and is associated with rising incidence and mortality rates (1, 2). The unsatisfactory outcomes emphasize the necessity for developing therapeutic targets to combat the progression of ICC, underscoring the importance of a deeper understanding of ICC pathogenesis.

Tumor-associated macrophages (TAMs) are crucial cellular components of the tumor microenvironment (TME) and play a significant role in regulating processes such as tumor growth, metastasis, and drug resistance (3). TAMs can polarize into two states, M1 and M2, depending on the specific environmental conditions (4). In various cancers, including ICC, an increase in M2 macrophages is often linked to a poor prognosis (5, 6). Alterations in the intermediates of the tricarboxylic acid (TCA) cycle, as a significant consequence of tumor metabolic reprogramming, have been reported to play a key role in determining the function of TAMs (7). Fumarate, an intermediate product of the TCA cycle, has been found to promote the anti-inflammatory phenotype associated with M2 polarization of macrophages (8).

The tripartite motif containing (*TRIM*) family is a significant group of E3 ubiquitin ligases that mediates ubiquitination modifications. It has been reported to be closely associated with various biological processes, including tumor cell proliferation, metabolism, and immune responses. Multiple members of the *TRIM* family, including *TRIM3*, *TRIM5*, *MID1*, *TRIM21*, *TRIM27*, *TRIM32*, *TRIM44*, *TRIM47*, and *TRIM72*, have been found to be highly expressed in HCC tissue samples and are significantly associated with poor prognosis in HCC patients (9). This prompts us to focus on the *TRIM* family, particularly the members that have been reported to be associated with poor prognosis in HCC. In this study, we analyzed the gene expression profiles of tumor tissues and adjacent tissues from patients with ICC using mRNA sequencing to identify the key genes involved in ICC. *TRIM47*, a member of the *TRIM* family, was upregulated in the ICC tissues (log_2_ fold change [FC] >2, *p* < 0.001). TRIM47 has been reported to function as a RING-type E3 ubiquitin ligase and has been proposed as a molecular target for anticancer therapies. In breast cancer, TRIM47 has been shown to activate the NF-κB signaling pathway by promoting the polyubiquitination of PKC-ε, which enhances the malignant phenotype of the tumor (10). The upregulated expression of TRIM47 has also been reported to promote the ubiquitination and degradation of SMAD4, thereby facilitating the progression of colorectal cancer (11). It has been reported that TRIM47 promoted aerobic glycolysis based on the ubiquitination pathway in pancreatic cancer (12). However, the comprehensive mechanisms of TRIM47 in ICC remain unclear.

Based on the function of TRIM47 in regulating the ubiquitination modification, to further elucidate the regulatory mechanism of TRIM47 in the progression of ICC, this study conducted an immunoprecipitation (IP) experiment using TRIM47 antibodies to isolate the binding partners of TRIM47, which were subsequently identified through mass spectrometry (MS) analysis. Functional enrichment analysis of the identified proteins revealed that fumarate hydratase (FH), a component of the TCA cycle, functions as a tumor suppressor and catalyzes the conversion of fumarate to succinate malic acid (13). Notably, the deletion or inactivation of FH results in the accumulation of fumarate. It has been reported that the expression of FH is decreased in ICC patients (14). Therefore, this study speculates that TRIM47 may regulate the expression of FH through the ubiquitination pathway, thereby affecting the polarization of TAMs mediated by fumarate, which further regulates the progression of ICC.

## Results

### Bioinformatics analysis

**Figure 1:**
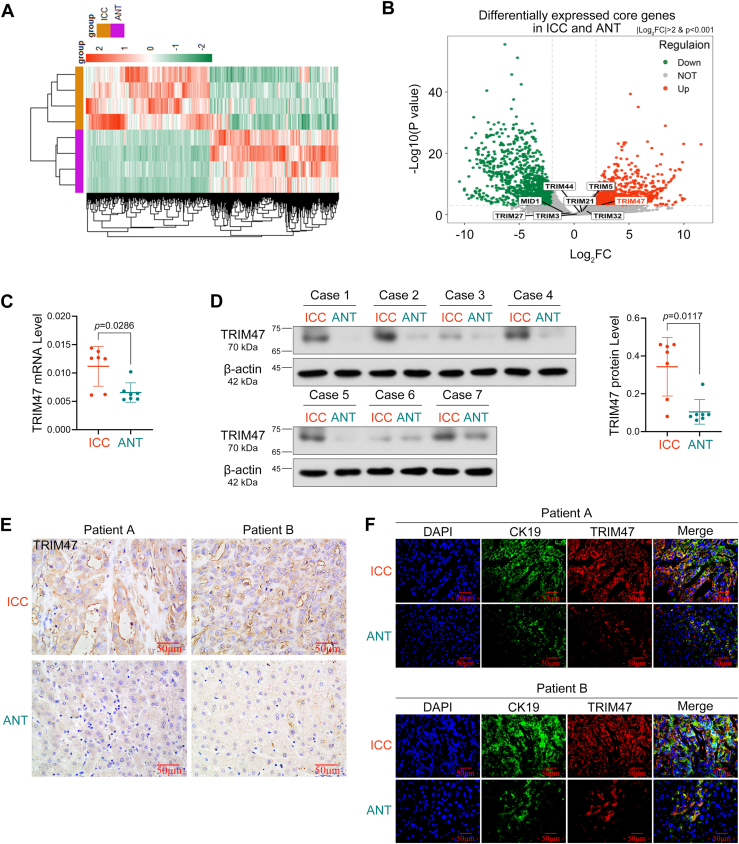
**TRIM47 expression is enhanced in patients with ICC and indicates a poor prognosis**. *A*, the heat map showing an overview of the DEGs in human ICC cases compared with adjacent normal tissues. *B*, the volcano map showing the expression of the interested TRIM family members in the RNA sequencing results. *C* and *D*, TRIM47 levels in ICC tumor and paired adjacent nontumor tissues. Statistical analysis was conducted using the Mann–Whitney test. *p* < 0.05 was considered statistically significant. *E*, immunohistochemical analysis of TRIM47 expression in human ICC paraffin sections. *F*, TRIM47 expression in CK19-positive tumor cells was detected using immunofluorescence staining. *p* < 0.05 was considered statistically significant. Data were presented as means ± SD. CK19, keratin 19; DEG, differentially expressed gene; ICC, intrahepatic cholangiocarcinoma; TRIM, tripartite motif containing; TRIM47, tripartite motif containing 47.

The results of RNA sequencing analysis showed that there were significant differences in the gene expression profiles between the tumor tissues and normal tissues of ICC patients (Fig. 1*A*). Gene Ontology enrichment analysis revealed that differentially expressed genes (DEGs) were involved in biological processes such as the TCA cycle, ubiquitination, and fumarate metabolism (Fig. S1*A*). Kyoto Encylopedia of Genes and Genomes (KEGG) enrichment analysis indicated that these DEGs may be associated with pathways related to carbon metabolism, amino acid metabolism, fatty acid metabolism, pyruvate metabolism, glycolysis, and the TCA cycle (Fig. S1*B*). Based on previous reports, members of the *TRIM* family—specifically *TRIM3*, *TRIM5*, *MID1*, *TRIM21*, *TRIM27*, *TRIM32*, *TRIM44*, *TRIM47*, and *TRIM72*—have been significantly associated with poor prognosis in HCC. We screened these key *TRIM* family members among the DEGs. Notably, we focused on *TRIM47*, the only differentially expressed member of the *TRIM* family linked to poor prognosis in ICC, which was significantly elevated in the tumor tissues of ICC patients (Fig. 1*B*).

### Increased TRIM47 expression predicts poor prognosis of ICC patients

The expression of TRIM47 in samples from patients with ICC was examined, revealing that TRIM47 levels were higher in tumor tissues compared with nontumor tissues (Fig. 1, *C*–*E*). The GEPIA online tool was utilized to analyze the relationship between *TRIM47* expression and patient survival in cholangiocarcinoma. High levels of *TRIM47* expression predict a poor prognosis and reduced disease-free survival within 60 months for patients diagnosed with cholangiocarcinoma (Fig. S2). Coexpression of TRIM47 and ICC marker CK19 was observed in patient samples, with both of them exhibiting elevated expression in tumor tissue samples, indicating the occurrence of ICC (Fig. 1*F*).

### TRIM47 promotes the proliferation of ICC cells and inhibits its apoptosis *in vitro*

**Figure 2:**
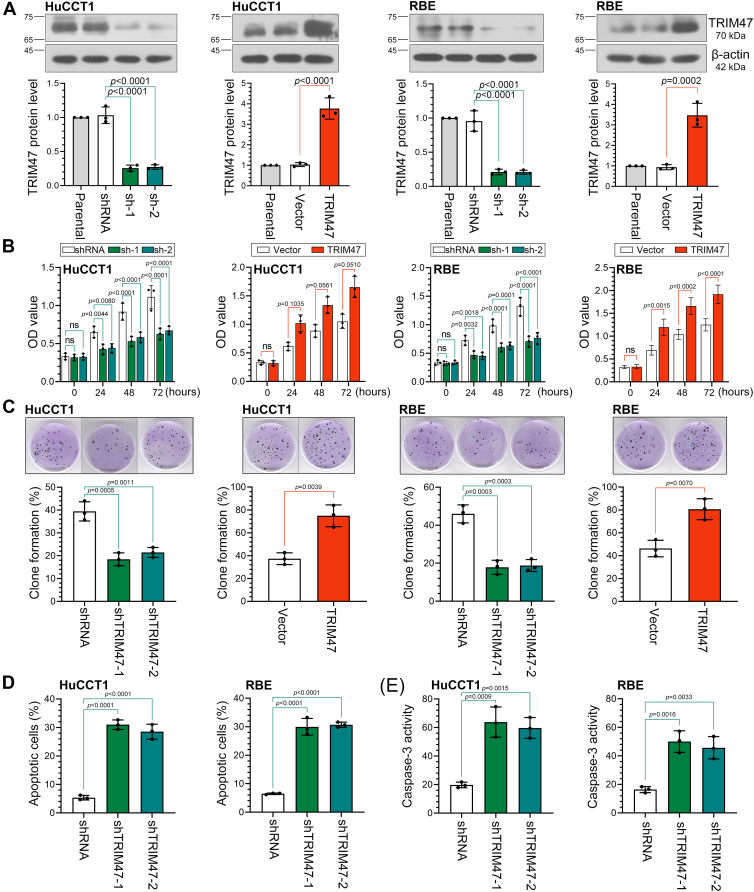
**TRIM47 promotes the proliferation of ICC cells and inhibits its apoptosis *in vitro***. *A*, Western blot analyses of TRIM47 expression in infected ICC cell lines. *B*, cell proliferation was measured using the Cell Counting Kit-8 assay. Statistical analysis was conducted using two-way analysis of variance. *C*, colony formation of ICC cells. Statistical analysis was conducted using the unpaired *t* test or one-way analysis of variance. *D*, apoptosis in ICC cells was detected using flow cytometry based on Annexin V staining. Statistical analysis was conducted using the one-way analysis of variance. *E*, the activity of caspase-3 was detected using the kit. Statistical analysis was conducted using the one-way analysis of variance. N = 3. *p* < 0.05 was considered statistically significant. Data were presented as means ± SD. Not significant, *p* > 0.05. ICC, intrahepatic cholangiocarcinoma; TRIM47, tripartite motif containing 47.

To elucidate the specific effects of TRIM47 on ICC, cell lines with overexpression and knockdown of TRIM47 were established in the ICC cell lines HuCCT1 and RBE, as demonstrated by Western blot analysis (Fig. 2*A*). Monitoring the proliferation of ICC cells over a 72-h period revealed that TRIM47 knockdown significantly inhibited cell proliferation, whereas TRIM47 overexpression enhanced it (Fig. 2*B*). In addition, TRIM47 knockdown reduced ICC cell colony formation, which was promoted by TRIM47 overexpression (Fig. 2*C*). Conversely, TRIM47 knockdown increased ICC cell apoptosis (Fig. 2*D*), a finding further corroborated by the measurement of caspase-3 activity (Fig. 2*E*).

### TRIM47 drives M2 polarization of TAMs

**Figure 3:**
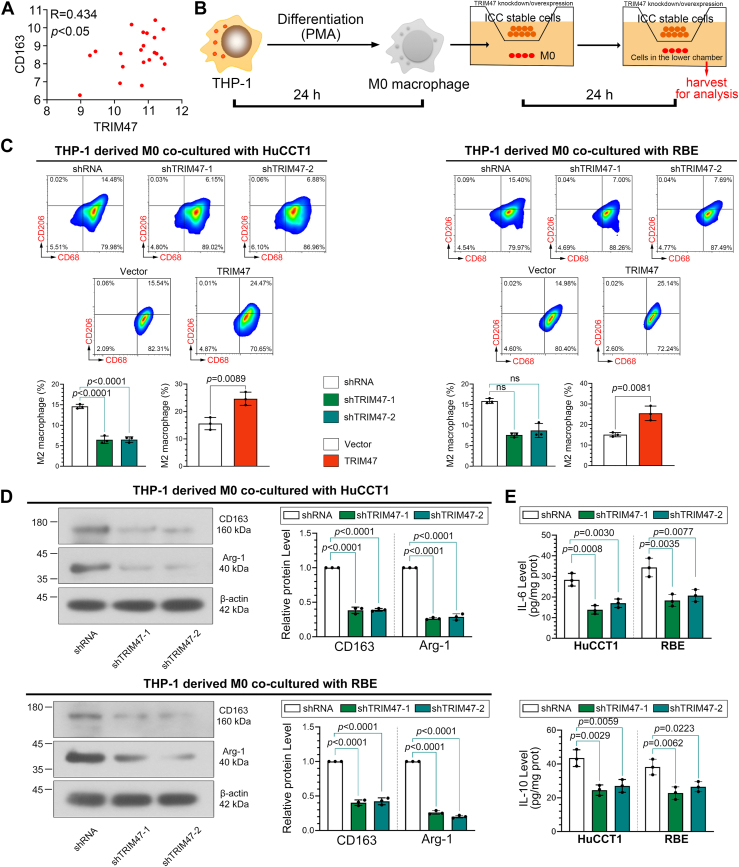
**TRIM47 drives M2 polarization of TAMs**. *A*, correlation analysis of the expression of TRIM47 and CD163 in the patient samples of cholangiocarcinoma derived from The Cancer Genome Atlas database. *B*, THP-1 cells were treated with PMA to induce M0 macrophage differentiation and were cocultured with indicated cells for 24 h. Cells in the lower chamber were harvested for analysis using flow cytometry. *C*, CD68^+^CD206^+^ TAMs were detected using flow cytometry. Statistical analysis was conducted using the one-way analysis of variance or Kruskal–Wallis test or unpaired *t* tests. *D*, the expression of CD163 and arginase-1 in the cells in the lower chamber. Statistical analysis was conducted using the one-way analysis of variance. *E*, the levels of interleukin (IL)-6 and IL-10 in the cells in the lower chamber. Statistical analysis was conducted using the one-way analysis of variance. N = 3. *p* < 0.05 was considered statistically significant. Data were presented as means ± SD. Not significant, *p* > 0.05. ICC, intrahepatic cholangiocarcinoma; TRIM47, tripartite motif containing 47.

The expression of TRIM47 and CD163 in the cholangiocarcinoma patient samples from The Cancer Genome Atlas database was positively correlated (Fig. 3*A*). To explore the function of TRIM47 in TME, we examined the levels of M2 macrophages in macrophages cocultured with ICC cells (Fig. 3*B*). Knockdown of TRIM47 led to a decrease in CD68+CD206+ cells in the cells cocultured with ICC cells (Fig. 3*C*) as well as a reduction in the expression of M2 polarization markers CD163 and arginase-1 (Fig. 3*D*). The contents of interleukin (IL)-6 and IL-10 in the cells cocultured with ICC cells also showed the same trend (Fig. 3*E*). However, overexpression of TRIM47 increased the level of M2 macrophages in the cells cocultured with ICC cells.

### The TME formed by TRIM47-silenced ICC cells is not conducive to the proliferation of ICC cells

**Figure 4:**
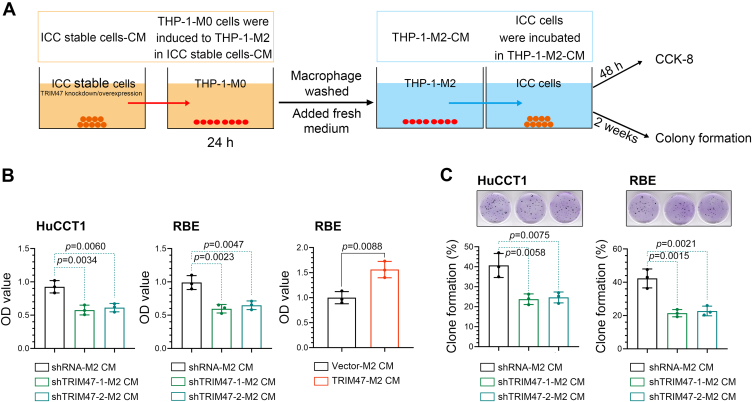
**The TME formed by TRIM47-silenced ICC cells is not conducive to the proliferation of ICC cells**. *A*, the conditioned medium from the THP-1-derived M2 macrophages educated by TRIM47-knockdown or overexpressed ICC cells was collected and used to treat ICC cells. *B*, cell proliferation of ICC cells was measured using the Cell Counting Kit-8 assay. *C*, colony formation of ICC cells. Statistical analysis was conducted using the one-way analysis of variance or unpaired *t* tests. N = 3. *p* < 0.05 was considered statistically significant. Data were presented as means ± SD. ICC, intrahepatic cholangiocarcinoma; TME, tumor microenvironment; TRIM47, tripartite motif containing 47.

Based on the observed promotion of M2 polarization of TAMs in the TME of TRIM47-overexpressed ICC cells, we further investigated the impact of the altered TME on ICC cells in return. The conditioned medium from TRIM47 knockdown or overexpression cells was collected and used to treat M0 macrophages for 24 h to induce their polarization into M2 macrophages. Subsequently, the conditioned medium from the M2 macrophages was collected and utilized to treat ICC cells (Fig. 4*A*). It was observed that the proliferation and clone formation of ICC cells were reduced when treated with conditioned medium from macrophages that had been educated by TRIM47-silenced ICC cells (Fig. 4, *B*–*C*). On the contrary, the ICC cells treated with conditioned medium from macrophages, which were educated by ICC cells overexpressing TRIM47, exhibited an increase in proliferation. This suggests that the TME of ICC cells, which were modified by TRIM47 knockdown, contributes to the inhibition of ICC cell proliferation.

### TRIM47 silencing inhibits M2 polarization of TAMs by reducing fumarate levels

**Figure 5:**
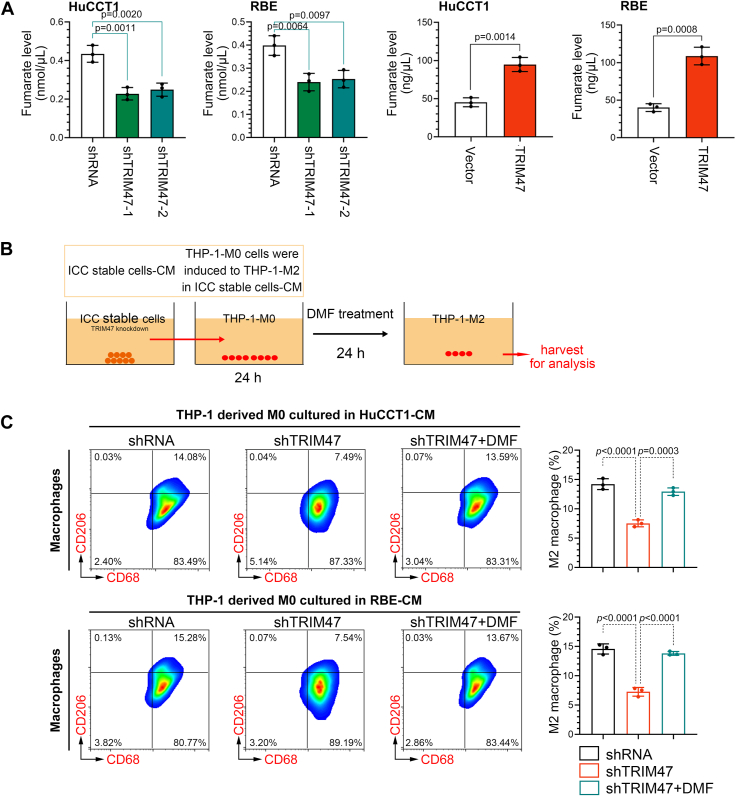
**TRIM47 silencing inhibits M2 polarization of TAMs by reducing fumarate level**. *A*, the content of fumarate in ICC cells. Statistical analysis was conducted using the one-way analysis of variance or unpaired *t* tests. *B*, the conditioned medium collected from the TRIM47-knockdown ICC cells was used to treat the THP-1-derived M0 macrophages for 24 h, and the DMF was added to treat the cells for an additional 24 h. Cells were harvested for analysis using flow cytometry. *C*, CD68^+^CD206^+^ TAMs were detected using flow cytometry. Statistical analysis was conducted using the one-way analysis of variance. N = 3. *p* < 0.05 was considered statistically significant. Data were presented as means ± SD. DMF, dimethyl fumarate; ICC, intrahepatic cholangiocarcinoma; TAM, tumor-associated macrophage; TRIM47, tripartite motif containing 47.

To verify the role of fumarate in the M2 polarization of TAMs, we examined the levels of fumarate in ICC cells. The results showed that knockdown and overexpression of TRIM47 inhibited and promoted the production of fumarate *in vivo* and *in vitro*, respectively (Fig. 5*A*). Subsequently, the THP-derived macrophages were cocultured with ICC cells and were treated using dimethyl fumarate (DMF), a derivative of a fumarate, to conduct a rescue experiment (Fig. 5*B*). The M2 polarization of macrophages was then examined. It was observed that TRIM47 knockdown inhibited the M2 polarization of TAMs, whereas DMF treatment reversed this effect (Fig. 5*C*).

### TRIM47 promotes tumorigenesis *in vivo*

**Figure 6:**
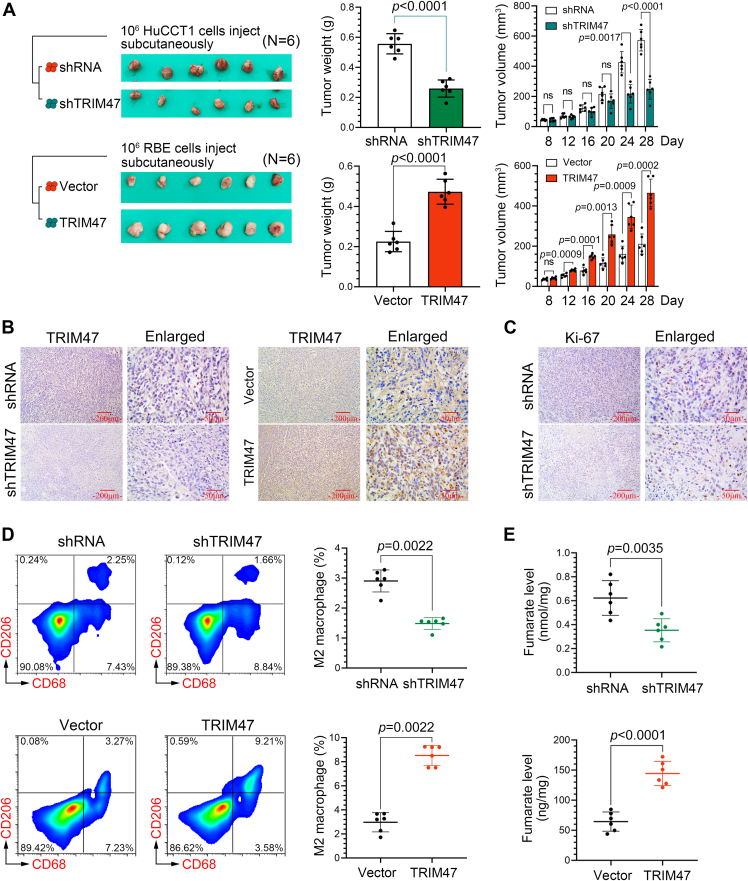
**TRIM47 promotes tumorigenesis *in vivo***. *A*, the tumor weight and volume of xenograft tumors were measured in the xenograft model. Statistical analysis was conducted using the two-way analysis of variance or unpaired *t* tests. Immunohistochemistry staining of TRIM47 (*B*) and Ki-67 (*C*) expression in tumor tissues. *D*, the percentage of CD68+CD206+ macrophages in tumor tissues was analyzed using flow cytometry. Statistical analysis was conducted using the Kruskal–Wallis test. *E*, the content of fumarate in tumor tissues. Statistical analysis was conducted using the unpaired *t* tests. N = 6. *p* < 0.05 was considered statistically significant. Data were presented as means ± SD. Not significant, *p* > 0.05. ICC, intrahepatic cholangiocarcinoma; TRIM47, tripartite motif containing 47.

Based on the findings from *in vitro* studies, we confirmed the impact of TRIM47 on the progression of ICC in a xenograft model. Compared with the control group, the growth of tumors formed by TRIM47-silenced ICC cells was significantly slowed; however, the tumors formed by TRIM47-overexpressed ICC cells exhibited accelerated growth (Fig. 6*A*). The successful knockdown and overexpression of TRIM47 was verified by examining its expression in the tumor tissues (Fig. 6*B*). The expression of Ki67 was reduced in the tumor tissues with TRIM47 knockdown, indicating that tumor proliferation was inhibited (Fig. 6*C*). Furthermore, silencing TRIM47 resulted in a decrease in CD68+CD206+ cells within the tumor tissues, whereas overexpression of TRIM47 led to an increase in CD68+CD206+ cells in the same tissues (Fig. 6*D*). In terms of fumarate level, TRIM47 silence reduced the fumarate level in tumor tissues. Conversely, the opposite effect was observed in tumor tissues where TRIM47 was overexpressed (Fig. 6*E*). These results are consistent with those observed in the *in vitro* experiments.

### FH is identified as an interacting protein of TRIM47 in ICC cells

**Figure 7:**
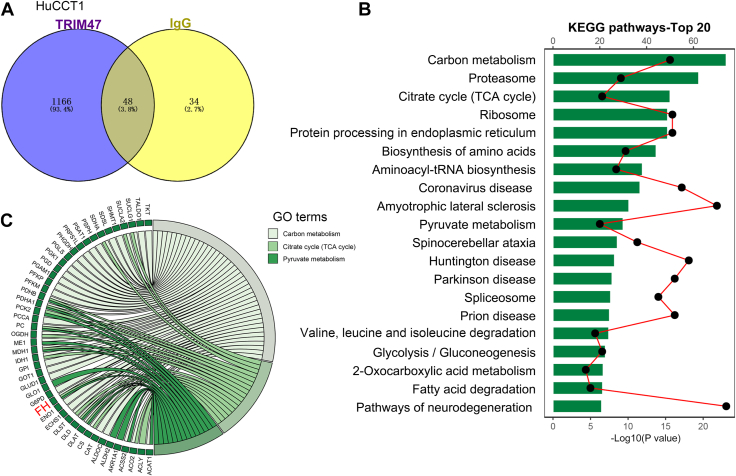
**FH is identified as an interacting protein of TRIM47 in ICC cells**. *A*, co-IP followed by MS identification was performed to detect the interacting protein of TRIM47 in ICC cells. *B*, enrichment analysis of Kyoto Encyclopedia of Genes and Genomes. *C*, chord plot of the relationship between the pathways and their corresponding genes. Co-IP, coimmunoprecipitation; FH, fumarate hydratase; ICC, intrahepatic cholangiocarcinoma; MS, mass spectrometry; TRIM47, tripartite motif containing 47.

To further investigate the role of TRIM67 in ICC, MS analysis was performed following co-IP using a TRIM67 antibody in HuCCT1 cells to identify the interacting proteins of TRIM47. A total of 1166 proteins were identified as specific interactors of TRIM47 (Fig. 7*A*). KEGG enrichment analysis revealed that the interacting proteins of TRIM47 might be involved in pathways related to carbon metabolism, proteasomes, TCA cycle, ribosomes, protein processing, amino acid biosynthesis, and pyruvate metabolism (Fig. 7*B*). In this study, we mainly focused on fumarate-related tumor cell metabolism. Therefore, we comprehensively analyzed the proteins enriched in carbon metabolism, TCA cycle, and the pyruvate metabolism pathway. Seven key proteins, involved in these metabolic pathways, were obtained (Fig. 7*C*). Through extensive literature review, we focused on FH, a key enzyme that catalyzes the metabolism of fumarate in the TCA cycle.

### TRIM47 negatively regulates FH expression through the ubiquitination-dependent pathway

**Figure 8:**
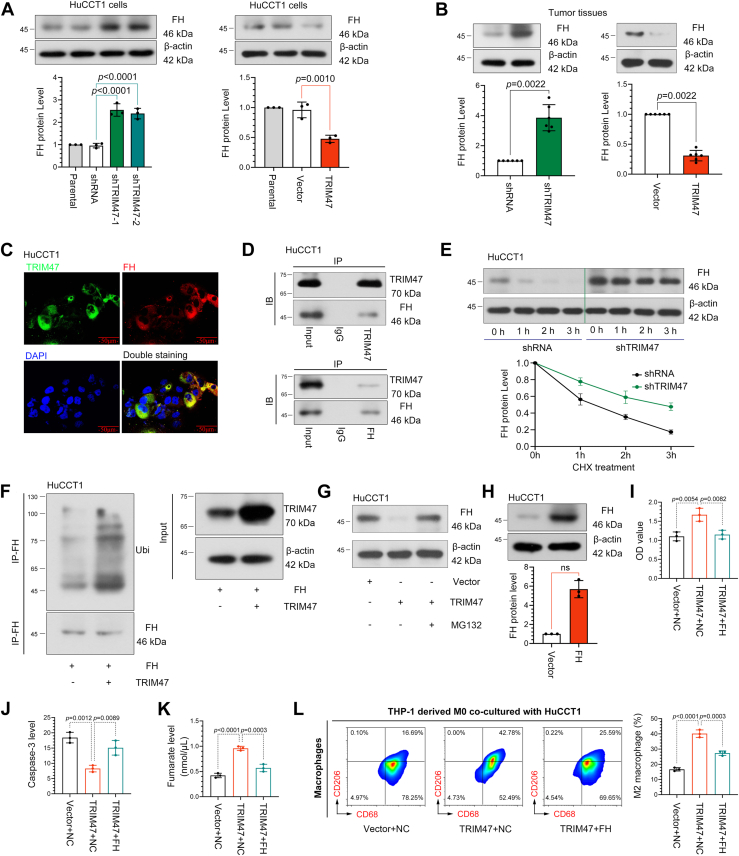
**TRIM47 promotes the progression of ICC through enhancing the ubiquitination-mediated degradation of FH**. FH expression in HuCCT1 cells (*A*) and tumor tissues (N = 6) (*B*). Statistical analysis was conducted using the one-way analysis of variance or Mann–Whitney test. *C*, the colocalization of TRIM47 (*green*) and FH (*red*) in one HuCCT1 cell was detected using immunofluorescence assays. *D*, co-IP was carried out using TRIM47, FH, or IgG antibodies, and specific binding proteins of TRIM47 and FH were analyzed using Western blotting. *E*, HuCCT1 cells with TRIM47 silence were treated with cycloheximide (200 mg/l) for the indicated time. Protein levels of FH were analyzed using Western blotting. *F*, the ubiquitination of FH in TRIM47-overexpressed HuCCT1 cells. *G*, protein levels of FH in the proteasome inhibitor MG132-treated HuCCT1 cells were analyzed using Western blotting. *H*, Western blot analyses of FH expression in infected ICC cell lines. Statistical analysis was conducted using the Mann–Whitney test. *I*, cell viability of ICC cells was measured using the Cell Counting Kit-8 assay. *J*, the activity of caspase-3 was detected using the kit. *K*, the content of fumarate in ICC cells. *L*, CD68+CD206+ macrophages in the THP-1 cells cocultured with ICC cells were analyzed using flow cytometry. Statistical analysis was conducted using the one-way analysis of variance. N = 3. *p* < 0.05 was considered statistically significant. Data were presented as means ± SD. Not significant, *p* > 0.05. Co-IP, coimmunoprecipitation; FH, fumarate hydratase; ICC, intrahepatic cholangiocarcinoma; TRIM47, tripartite motif containing 47.

TRIM47 was identified as a negative regulator of FH expression in ICC cells and tumor tissues (Fig. 8, *A* and *B*). Colocalization of endogenous TRIM47 and FH in HuCCT1 cells was observed (Fig. 8*C*). The interaction between TRIM47 and FH was confirmed through Western blotting following a co-IP experiment (Fig. 8*D*). Knockdown of TRIM47 increased the protein half-life of FH from 1.33 h to 2.25 h, thereby inhibiting the degradation of the FH protein (Fig. 8*E*). An increase in the ubiquitination level of FH upon the overexpression of TRIM47 was observed. In addition, the expression of FH, which was inhibited by TRIM47, increased following treatment with the proteasome inhibitor MG132 (Fig. 8, F–G).

### TRIM47 silencing inhibits the progression of ICC by enhancing the expression of FH proteins

To examine whether FH mediates the regulation of TRIM47 on ICC, FH was forcibly expressed in ICC cells using a lentiviral system (Fig. 8*H*). The results of the rescue experiment demonstrated that FH overexpression abolished the effect of TRIM47 overexpression on ICC cells, including inhibiting cell viability (Fig. 8*I*), increasing caspase-3 activity (Fig. 8*J*), reducing fumarate level (Fig. 8*K*), and inhibiting M2 macrophage of TAMs (Fig. 8L).

## Discussion

TRIM47 has been reported to be highly expressed in various cancers and to play a procancer role. This study demonstrates that TRIM47, elevated in ICC, drives the M2 polarization of TAMs by enhancing the ubiquitination of FH and increasing fumarate level, thereby promoting ICC progress.

Many studies outside ICC have shown that TRIM47 promotes tumor cell proliferation and inhibits tumor cell apoptosis (11, 15, 16). We reported that TRIM47 plays a consistent role in ICC as demonstrated in previous studies. This includes enhancing cell proliferation, promoting clone formation, and reducing apoptosis. These evidences describe the direct effect of TRIM47 on the phenotype of ICC cells. However, since it is very difficult for us to obtain a sufficient number of patient samples in a short period, the correlation between TRIM47 and the clinical characteristics of ICC patients was not analyzed. We are aware of this limitation and are working to address it. If we can collect sufficient samples of ICC patients in the future, the clinical characteristics of TRIM47 and ICC patients will be further discussed. In addition, although this study identified TRIM47 as a therapeutic target for ICC, at the clinical level, methods to inhibit TRIM47, such as targeted drugs, require further in-depth exploration and experimental validation, particularly concerning toxicity in normal cells.

What cannot be ignored is TRIM47 has been extensively studied in cancer as an E3 ubiquitin ligase that enhances the ubiquitination and degradation of substrate proteins. The ubiquitination and degradation of proteins, such as FOXO1, SMAD4, P53, and XAF1, have been reported to mediate the malignant behavior of TRIM47 in promoting various cancers, including glioma, colon cancer, renal cell carcinoma, and squamous cell carcinoma of the head and neck (11, 17, 18, 19). In this study, the specific binding protein of TRIM47 was isolated using a co-IP experiment and identified through MS. The results suggest that FH may be a potential target protein of TRIM47. We focus on FH because of its significant role in catalyzing fumaric acid metabolism within the TCA cycle (20). Overexpression of FH has been reported to inhibit the progression of breast cancer and endometrial cancer (21, 22). This study found that FH overexpression reduced the proliferation of ICC cells and induced apoptosis, which is consistent with the known functions of FH.

In addition to the direct effect on the malignant behavior of ICC cells, this study also elucidated the regulation of TRIM47 on TAMs. It was found that fumarate, the catalytic substrate of FH, was positively regulated by TRIM47. DMF treatment drives the M2 polarization of TAMs by increasing the level of fumarate, which reverses the effect of TRIM47 knockdown. The overexpression of FH was observed to increase the level of fumarate, and it also reversed the effect of TRIM47 on the M2 polarization of TAMs in the rescue experiment. This evidence indicates that the M2 polarization of TAMs driven by TRIM47 is mediated by FH and is dependent on the catalytic function of FH for fumarate metabolism. The effect of FH on the M2 polarization of TAMs may be attributed to its regulation of the TME. It has been reported that in hereditary leiomyomatosis and renal cell carcinoma associated with FH deficiency, there is a significant accumulation of fumarate, leading tumor cells to favor aerobic glycolysis (23). It is well established that aerobic glycolysis can create a microenvironment conducive to tumor development because of the resulting accumulation of lactic acid (24). It is undeniable that changes in the TME may also affect other immune cells. Studies have shown that lactate treatment inhibits the proliferation of T cells (25) and the cytolytic function of NK cells *in vitro* (26). Knockdown of lactate dehydrogenase in breast cancer cells has been reported to enhance T-cell–mediated tumor immunity (27). Therefore, there is a possibility that TRIM47 affects immune cells other than macrophages. The research focused on the effects of TRIM47 overexpression or knockdown on the M2 polarization of TAMs. Therefore, BALB/c nude mice, which are T-cell deficient, were used for *in vivo* study. Studies have shown that T cells assist macrophages in acquiring the M1 tumor–killing phenotype (28). Furthermore, HHLA2 has been identified as an inhibitor of T-cell activation and proliferation, and it also suppresses the secretion of T-cell–related cytokines (29, 30). HHLA2 deficiency has been reported to inhibit the M2 polarization of THP-1 macrophages (31). It can be speculated that the absence of T cells created an *in vivo* environment conducive to the polarization of macrophages toward the M2 phenotype. Even so, the consistent *in vivo* environment controls for this variable. Therefore, the use of BALB/c nude mice with T-cell deficiency does not affect the validity of the observational results in this study. On the contrary, it eliminates the interference of the immune effect of T cells.

Although this study was based on the role of FH in tumor cell metabolism, it cannot be denied that FH may regulate some signal pathway. Studies have shown that FH inhibits the metastasis of non–small cell lung cancer by suppressing the AMP-activated protein kinase signal pathway (32). FH knockdown leads to an increase in the phosphorylation level of epidermal growth factor receptor, thereby promoting the proliferation and metastasis of endometrial cancer (21). It can be seen that the regulation of FH on cancer may involve multiple mechanisms.

In addition to FH, the KEGG enrichment analysis results following MS identification indicate that PDHB, PDHA1, PC, MDH1, DLD, and DLAT are also key proteins involved in carbon metabolism, the TCA cycle, and pyruvate metabolism in cancers, including HCC, ICC, breast cancer, and pancreatic ductal cancer (33, 34, 35, 36, 37). Therefore, these tumor metabolic–related proteins may regulate ICC cell metabolic reprogramming through direct or indirect interactions with TRIM47, which is worthy of in-depth investigation in future work.

In conclusion, this study elucidates the mechanism by which TRIM47 facilitates the development of ICC by inhibiting the expression of FH through the ubiquitination pathway. This inhibition results in elevated levels of fumarate, which in turn promotes the M2 polarization of TAMs. The findings highlight significant molecular mechanisms underlying ICC development from the perspective of TME, thereby offering a preclinical strategy for targeted therapy of ICC.

## Experimental procedures

### Clinical samples, RNA sequencing, and data analysis

Seven pairs of the tumor and adjacent nontumor tissues were collected from patients diagnosed with ICC. Written informed consent was obtained from all participants. This study was conducted in accordance with the Declaration of Helsinki and was approved by the Medical Ethics Committee of Shengjing Hospital of China Medical University (approval number: 2024PS1302K).

RNA sequencing was performed by Novogene Co, Ltd. In brief, total RNA was isolated from the patient samples using TRIzol reagent (catalog no.: RP1001; BioTeke) and was fragmentated using fragmentation buffer. The RNA fragments were used as templates for reverse transcription to synthesize complementary DNA (cDNA). The library was constructed using the Invitrogen SuperScript II Reverse Transcriptase (Invitrogen). RNA sequencing analysis was performed using the Illumina high-throughput sequencing platform NovaSeq 6000.

DEGs were identified using the limma R package (23) with log_2_FC >2 or <−2 and *p* value <0.001, followed by Gene Ontology and KEGG enrichment analysis.

### Survival analysis

The correlation between the expression of TRIM47 and the disease-free survival period of ICC patients was analyzed using the GEPIA online website based on The Cancer Genome Atlas database.

### Lentivirus preparation

The coding sequences of TRIM47 and FH were cloned into the pLVX-IRES-puro plasmid to construct the TRIM47 and FH overexpression constructs, respectively. Two shRNA sequences targeting TRIM47 (5′-GCAGCTGTTTGGAACCAAAGG-3′ and 5′-GGAGCTCAGCTTCACCAAATC-3′) were synthesized and cloned into the pLVX-shRNA1 plasmid to construct the TRIM47 knockdown construct. Recombinant plasmids carrying exogenous gene fragments were transfected into 293T cells and cultured at 37 °C and 5% CO_2_. At 48 h and 72 h after infection, the cell supernatants were collected and filtered to obtain recombinant lentivirus particles.

### Cell infection and treatment

Human ICC cell lines HuCCT1 and RBE were obtained from iCell Bioscience and cultured in RPMI1640 medium. The cell lines have been identified using the short tandem repeat profiling, and there is no mycoplasma infection. The cells were infected with the prepared lentivirus. After 48 h, the cells were treated with 1.5 μg/ml puromycin to screen for the stably infected cell strain. In brief, control and lentivirus-infected cells were cultured in medium containing puromycin, with the medium replaced every 2 days. Once all control cells had died, the surviving lentivirus-infected cells were collected. These cells were then seeded at a density of one cell per well into a 96-well culture plate using the limiting dilution method. Wells containing a single adherent cell, confirmed by microscopic observation, were labeled, and the culture was continued until the cells expanded. Subsequently, monoclonal cells were collected and passaged to establish stably infected cell lines. The expression of the target gene was examined using Western blot.

To determine the protein half-life, the infected cells were treated with the protein synthesis inhibitor cycloheximide (100 mg/ml, catalog no.: C112766; Aladdin) for 0, 1, 2, and 3 h. The protein of FH was determined using Western blotting.

### Cell coculture

In this study, the THP-1 cell lines or human peripheral blood mononuclear cells (PBMCs), obtained from iCell Bioscience were used to induce differentiation into macrophages. The THP-1 cell line and PBMC have been identified using the short tandem repeat profiling, and there is no mycoplasma infection. PBMCs were treated with 100 ng/ml macrophage colony stimulating factor (MedChemExpress) to induce their differentiation into M0 macrophages. The THP-1 cell line was isolated from the peripheral blood of a male patient with monocytic leukemia. THP-1 cells serve as a suitable substitute for wildtype macrophages because of their homogeneous genetic background, which minimizes variation in cell phenotypes and ensures the stability and reproducibility of experimental results. THP-1 cells were treated with 100 nM phorbol-12-myristate-13-acetate (catalog no.: R32414; Yuanye) for 24 h to induce their differentiation into M0 macrophages. Induced M0 macrophages were then seeded into the lower chamber of the transwell inserts. ICC cells were seeded into the upper chamber. The cells were cocultured for 24 h, and the cells in the lower chamber were collected for further analysis.

Conditioned culture media harvested from the stable infected ICC cells were used to treat M0 macrophages for 24 h to obtain M2 macrophages. These M2 macrophages were then cultured for an additional 48 h to collect the cell supernatant, which served as the conditioned medium for treating ICC cells that did not receive any exogenous gene transduction. The ICC cells were subsequently collected, and their cell proliferation and clone formation ability were assessed.

Conditioned culture media harvested from the stable infected ICC cells were used to treat M0 macrophages for 24 h to obtain M2 macrophages. These M2 macrophages were then treated with DMF for an additional 24 h, and the cells were collected for further analysis.

### Cell proliferation

Cell proliferation was measured by Cell Counting Kit-8 Proliferation Assay Kit (catalog no.: KGA9305; KeyGen Biotech) according to the manufacturer’s instructions. ICC cells were seeded into a 96-well plate at a density of 5 × 10^3^ cells per well. The cells were treated in accordance with the established protocol. The absorbance at 450 nM was detected using a microplate reader (BioTek).

### Clone formation

The clone formation assay was performed to detect cell proliferation. Briefly, cells were seeded into plates at a density of 300 cells per well and cultured for 2 weeks. Cells were fixed and stained with crystal violet dye. The number of colonies formed was counted using an inverted microscope (Olympus).

### Cell apoptosis

Cell apoptosis was measured using an Annexin V-FITC/Propidium Iodide Cell Apoptosis Detection Kit (catalog no.: KGA1102; KeyGen Biotech). Cells were stained with Annexin V-FITC and propidium iodide and then subjected to flow cytometry. Apoptotic cells were quantified using FlowJo software (Becton, Dickinson and Company).

### Caspase-3 activity

A Caspase 3 Activity Assay Kit (catalog no.: C1116; Beyotime) was used to detect caspase-3 activity by evaluating the content of *p*-nitroaniline, as previously reported (25). The cells were lysed in lysis buffer and incubated with Ac-DEVD-*p*-nitroaniline for 1 h at 37 °C. The absorbance at 405 nm was measured using a microplate reader.

### Fumarate level

The levels of fumarate were measured using the fumarate assay kit (catalog no.: F486219; Aladdin; catalog no.: MAK060) according to the instructions provided by the manufacturers.

### Inflammatory factors

The levels of inflammatory factors were detected using the kits based on the enzyme-linked immunosorbent assay. Human IL-10 and IL-6 ELISA kits (catalog nos.: EH0201, EH0173; FineBio) were used to assess the levels of IL-10 and IL-6, respectively.

### Tumor xenotransplantation

BALB/c male nude mice (6–8 weeks old), which are T-cell deficient, were housed in the specific additions (22 ± 1 °C, 45–55% humidity, 12/12-h light–dark cycle). Stable HuCCT1 cells infected with the lentivirus (approximately 1 × 10^6^) were injected into mice (six mice/group). Tumor sizes were monitored every 4 days. Mice were asphyxiated by CO_2_ inhalation after 28 days. Tumor samples were collected for further analysis. The animal experiment was approved by the Medical Ethics Committee of Shengjing Hospital of China Medical University (approval number: 2024PS1350K).

### Immunohistochemistry and immunofluorescence

Tissue samples were embedded in paraffin and cut into 5-μm sections, which were dewaxed and blocked with 1% bovine serum albumin (BSA). Sections were then hybridized with primary antibodies recognizing TRIM47 (catalog no.: 26885-1-AP; Proteintech Group, Inc) and Ki67 (catalog no.: AF0198; Affinity) at 4 °C for 12 h. The sections were processed with horseradish peroxidase–labeled secondary antibodies for 1 h and detected using a 3,3′-diaminobenzidine solution. Following counterstaining with hematoxylin and mounting, the images were captured using an optical microscope (Olympus).

For immunofluorescence in tissues, the sections were blocked with 1% BSA and then incubated with the primary antibody against CK19 (catalog no.: Ab20210; Abcam) and TRIM47 (catalog no.: 26885-1-AP; Proteintech Group, Inc) at 4 °C for 12 h. For immunofluorescence in cells, the cells were seeded on the slides and fixed in 4% paraformaldehyde for 15 min. The cells were blocked with 1% BSA and then incubated with the primary antibody against FH (catalog no.: Sc-393992; Santa Cruz Biotechnology, Inc) and TRIM47 (catalog no.: 26885-1-AP; Proteintech Group, Inc) at 4 °C for 12 h. The samples were incubated with the corresponding secondary antibody for 1 h. Nuclei were stained with 4',6-diamidino-2-phenylindole. The slides were photographed under a fluorescence microscope (Olympus).

### Flow cytometry

The tumor tissue was digested with collagenase at 37 °C, then homogenized, and centrifuged to eliminate connective tissue and obtain the cell suspension. The cell suspension was treated with red blood cell lysis buffer and centrifuged again to remove any residual red blood cell debris. The cells were resuspended in PBS buffer and incubated with the CD206 antibody for 30 min. Following centrifugation, the precipitate was collected. The precipitate was then treated with a membrane-disrupting solution for 30 min and subsequently incubated with the CD68 antibody for an additional 30 min. Flow cytometry was employed for analysis.

### Co-IP–MS

Total proteins were extracted from HuCCT1 cells using the IP buffer (20 mM Tris, pH 8.0, 150 mM NaCl, 1 mM EDTA, and PMSF). Co-IP was performed with the TRIM47 antibodies and Protein A/G-agarose beads according to the manufacturer’s instructions. The interacting proteins of TRIM47 were subjected to SDS-PAGE, and the protein bands were enzymatically hydrolyzed using trypsin, desalinated with a C18 chromatographic column, freeze-dried, and prepared for MS analysis. The MS analysis was conducted using a Q Exactive HF-X mass spectrometer equipped with a Nanospray Flex (electrospray ionization) ion source. The data were analyzed with the R package limma. Log_2_FC >1 and *p* value <0.05 were used for identifying candidate proteins.

### Real-time quantitative PCR

Total RNA was extracted using TRIzol reagent (catalog no.: RP1001; BioTeke), and the cDNA was obtained through reverse transcription using the TransScript All-in-One First-Strand cDNA Synthesis SuperMix (MD80101; Magen Biotech). Real-time quantitative PCR was performed using the SYBR Green (catalog no.: SR4110; Solarbio) in the system containing cDNA, primers, and 2× Taq PCR Master Mix (catalog no.: PC1150; Solarbio). The mRNA expression was quantified relative to β-actin expression using the 2-^ΔCt^ and 2-^ΔΔCt^ methods. The sequences of the primers are as follows. *Homo* *TRIM47* positive-sense, 5′-GCTTCTCCGTCTGGTTTCA-3′ and antisense, 5′-CATCTTGCCGTCCCGTA-3′.

### Western blot

Proteins were isolated using cell lysis buffer (catalog no.: P0013C; Beyotime) and qualified using a Bicinchoninic Acid Assay detection kit (catalog no.: P0011; Beyotime) following the manufacturer’s protocol. Equal amounts of protein were loaded on the SDS-PAGE to separate the protein bands. The separate proteins were transferred onto a polyvinylidene fluoride membrane and blocked with QuickBlock Western Blocking Buffer (catalog no.: P0239; Beyotime) for 1 h. The membrane was incubated with specific primary antibodies against TRIM47 (catalog no.: 26885-1-AP; Proteintech Group, Inc), FH (catalog no.: sc-393992; Santa Cruz Biotechnology, Inc), CD163 (catalog no.: A8383; Abclonal), arginase-1 (catalog no.: A1847; Abclonal), and Ubi (catalog no.: 10201-2-AP; Proteintech Group, Inc) at 4 °C for 12 h, followed by washing and incubation with secondary antibodies for 45 min. Protein bands were developed using an enhanced chemiluminescence reagent (catalog no.: P0018; Beyotime), and the gray value was analyzed using Gel-Pro-Analyzer software (Media Cybernetics, Inc).

### Statistical analysis

Statistical analyses were conducted using GraphPad Prism (version 8.0; GraphPad Software). Comparisons between two groups were performed using either Student’s *t* test or the Mann–Whitney *U* test. For comparisons involving more than two groups, one-way or two-way analysis of variance was utilized, followed by Tukey’s post hoc test. For the comparison of multiple groups of data that do not follow a normal distribution, the Kruskal–Wallis test was employed, based on Dunn’s post hoc test. *p* < 0.05 was considered statistically significant. Data are presented as means ± SD.

## Data availability

The data that support the findings of this study are available from the corresponding author upon reasonable request.

## Supporting information

This article contains supporting information.

## Conflict of interests

The authors declare that they have no conflicts of interest with the contents of this article.
