# Supplementary material for: E3 ubiquitin ligase TRIM47 promotes intrahepatic cholangiocarcinoma progression by ubiquitinating fumarate hydratase and modulating macrophage polarization
Source: J Biol Chem. 2025 Dec 9;302(1):111035. doi: 10.1016/j.jbc.2025.111035 (PMC12804375; doi:10.1016/j.jbc.2025.111035)
Supplement: Supplementary Material 1 [file mmc1.docx]

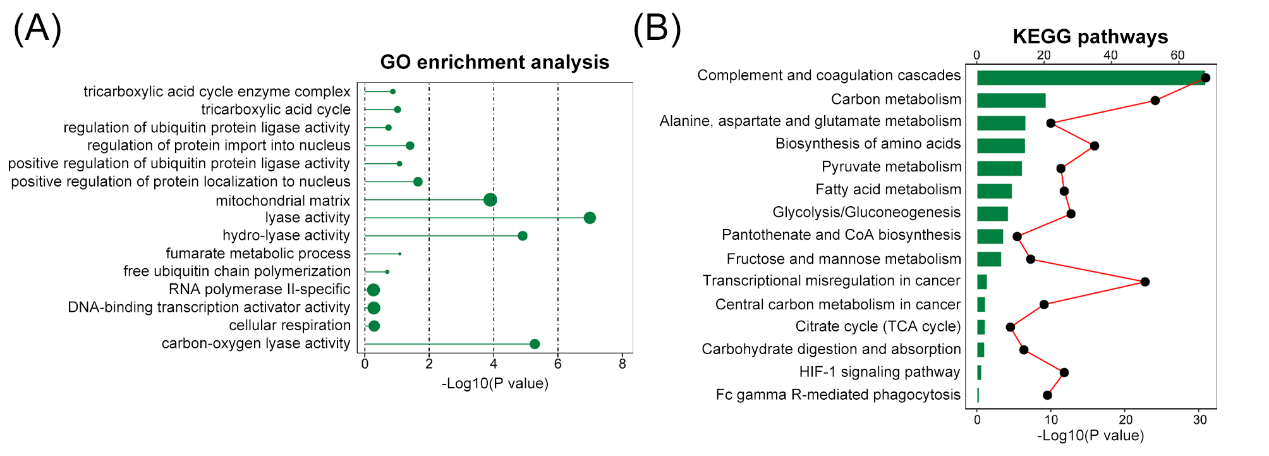


**Fig.S1** Enrichment analysis of Gene Ontology (A) and Kyoto Encyclopedia of Genes and Genomes (B).


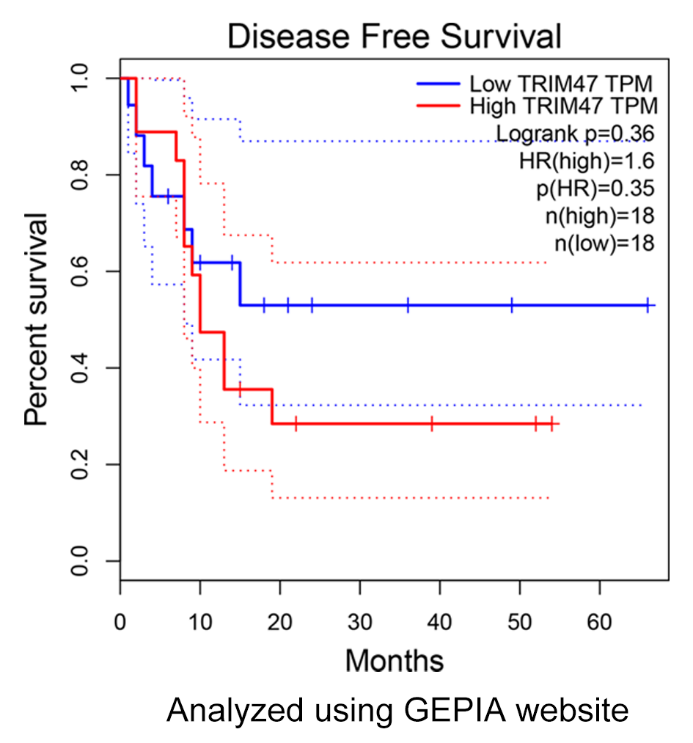


**Fig.S2** Kaplan–Meier analysis of the correlation between TRIM47 expression and disease-free survival of ICC patients using the Kaplan–Meier Plotter. ICC, intrahepatic cholangiocarcinoma; TRIM47, tripartite motif containing 47.


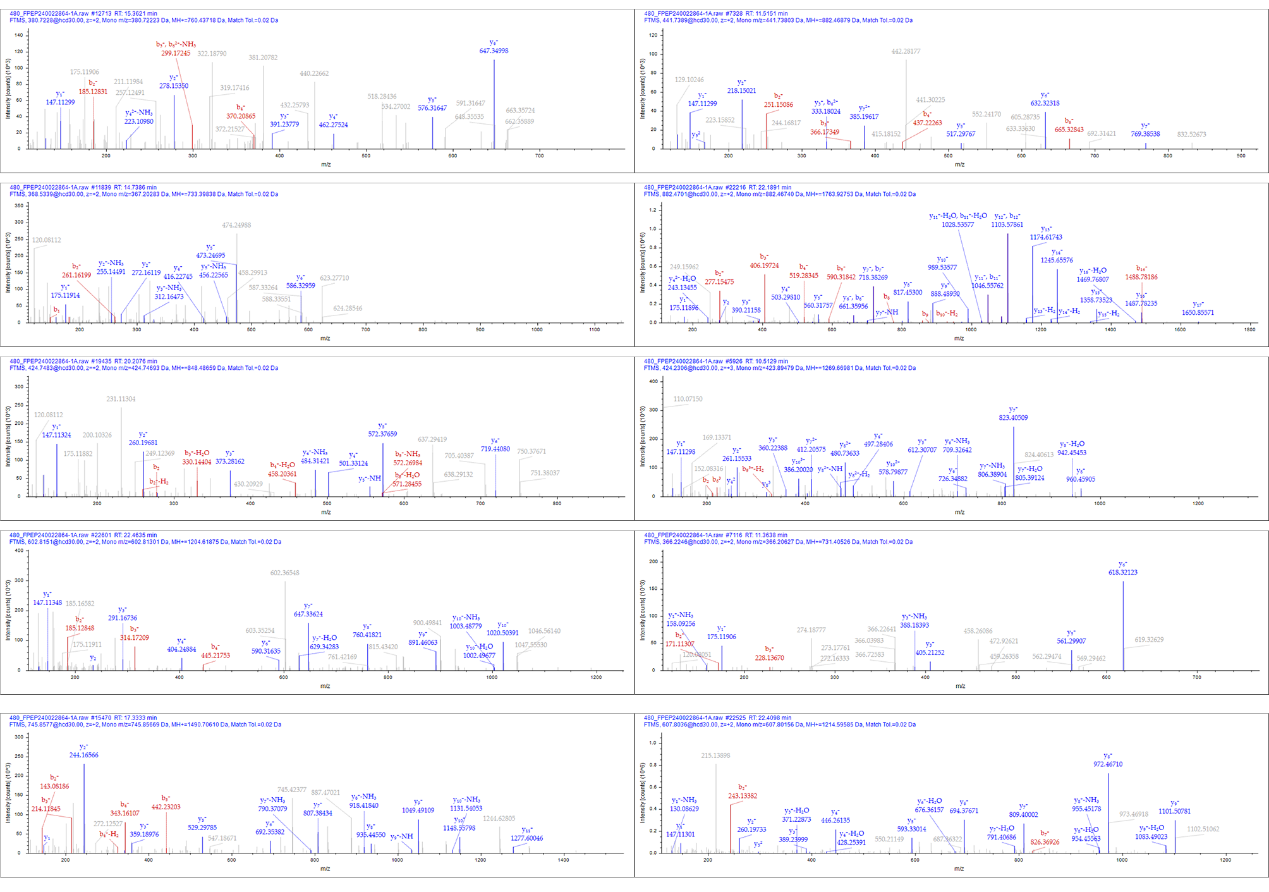


**Fig.S3** The representative peptide fragments peaks of fumarate hydratase was identified using mass spectrometry.


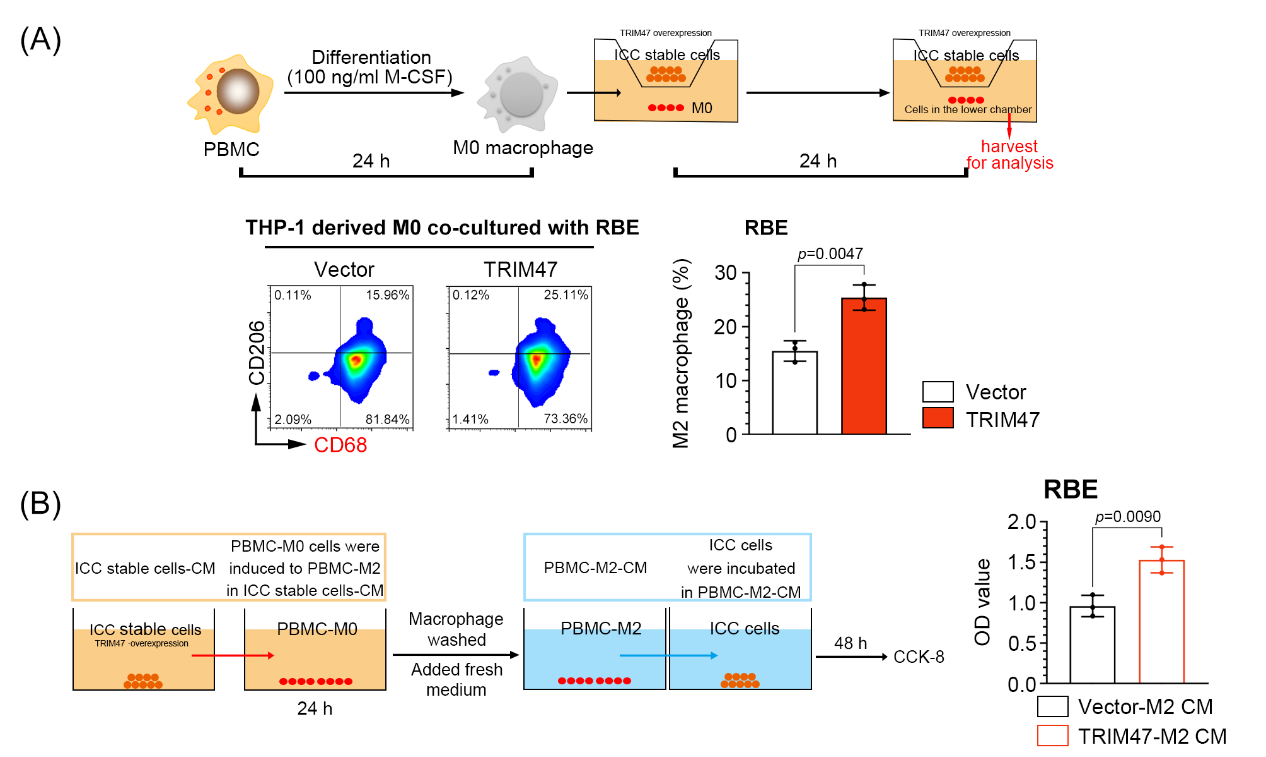


**Fig.S4** (A) Human peripheral blood mononuclear cells (PBMC) were treated with macrophage colony stimulating factor to induce M0 macrophages differentiation, and were co-cultured with indicated cells for 24 h. Cells in the lower chamber were harvested for analysis using flow cytometry. CD68^+^CD206^+^ TAMs were detected using flow cytometry. (B) The conditioned medium from the PBMC-derived M2 macrophages educated by TRIM47-knocked down or overexpressed ICC cells was collected, and used to treat ICC cells. Cell proliferation of ICC cells was measured using the CCK-8 assay. Statistical analysis was conducted using the unpaired t-tests. N=3. P < 0.05 was considered statistically significant. Data were presented as means ± SD. ICC, intrahepatic cholangiocarcinoma; TRIM47, tripartite motif containing 47.
